# Evaluating the potential of ChatGPT as an educational decision-support tool for hemodialysis decision-making in nephrology training

**DOI:** 10.3389/fmed.2026.1909864

**Published:** 2026-08-13

**Authors:** Esin Avsar Kucukkurt, Feyza Bora, Hasan Sozel, Dilek Yapar

**Affiliations:** 1 Department of Internal Medicine, Faculty of Medicine, Akdeniz University, Antalya, Türkiye; 2 Division of Nephrology, Department of Internal Medicine, Faculty of Medicine, Akdeniz University, Antalya, Türkiye; 3 Institute of Health Sciences, Department of Medical Informatics, Akdeniz University, Antalya, Türkiye

**Keywords:** artificial intelligence, ChatGPT, clinical decision support, hemodialysis, large language models, medical education, nephrology

## Abstract

**Background:**

This study aimed to evaluate the performance of ChatGPT in identifying hemodialysis (HD) indications from authentic nephrology consultation notes and to compare its recommendations with both real-world clinical decisions and expert nephrologist consensus.

**Methods:**

This exploratory observational study included 22 anonymized nephrology consultation notes from routine inpatient care at a tertiary care university hospital. Each note was independently evaluated by ChatGPT 5.4 using a standardized zero-shot prompt. The same notes were independently reviewed by three blinded senior academic nephrologists. The majority-vote consensus among these nephrologists was defined as the primary reference standard. Real-world decisions documented by nephrology fellows were evaluated as a secondary comparator. Agreement was assessed using Cohen’s kappa coefficient, and inter-rater reliability among nephrologists was evaluated using Fleiss’ kappa.

**Results:**

Expert consensus classified eight of 22 cases (40.9%) as requiring hemodialysis and 13 (59.1%) as not requiring HD. Inter-rater agreement among the nephrologists was excellent (Fleiss’ *κ* = 0.814, *p* < 0.001). ChatGPT and real-world clinical decisions agreed in 18 of 22 cases (81.8%; *κ* = 0.633, *p* = 0.003), whereas ChatGPT and expert consensus agreed in 15 of 22 cases (68.2%; *κ* = 0.374, *p* = 0.069). Expert consensus and real-world clinical decisions agreed in 17 of 22 cases (77.3%; *κ* = 0.553, *p* = 0.007). Among the nine expert-defined HD cases, ChatGPT agreed in seven cases and demonstrated exact indication-level agreement in four cases. Among the 10 cases classified as HD by both ChatGPT and real-world clinical decisions, exact indication-level agreement was observed in four cases. Discrepancies were most commonly observed in cases involving metabolic acidosis, volume overload, and oliguria/anuria.

**Conclusion:**

These exploratory findings suggest that ChatGPT may align more closely with nephrology fellows’ decision-making than with senior nephrologists’ judgments. The observed discrepancies may reflect differences in how clinical findings were interpreted and incorporated into the overall clinical context. Although ChatGPT cannot replace expert clinical judgment, it may have potential as an educational tool for trainees, encouraging systematic evaluation of HD indications and structured clinical reasoning. Larger, prospective, multicenter studies are needed to confirm these findings and evaluate ChatGPT’s educational impact in nephrology training.

## 1 Introduction

In recent years, artificial intelligence (AI) and large language models (LLMs) have shown considerable potential as clinical decision-support tools in healthcare. AI and machine learning algorithms have achieved high levels of accuracy in several nephrology-related applications, including the early detection of chronic kidney disease, prediction of acute kidney injury, and estimation of complication risk in patients undergoing dialysis or kidney transplantation (1–3). Similarly, LLMs such as ChatGPT have been reported to perform at a level comparable to specialist physicians in selected nephrology tasks, including consultation triage, continuous renal replacement therapy (CRRT) alarm management, and interpretation of laboratory data (4, 5). However, the clinical decision-making and triage performance of LLMs appears to vary substantially across different domains (6, 7). Previous studies have shown that AI models may demonstrate limited agreement in complex guideline-based treatment decisions, may misinterpret clinical thresholds, and in some cases may generate misleading recommendations that could pose risks to patient care (8, 9).

The decision to initiate hemodialysis in patients with acute kidney injury is a complex process that often requires the integration of laboratory findings, hemodynamic status, metabolic disturbances, comorbid conditions, and clinical judgment. In many tertiary care hospitals and university hospitals, the decision to initiate kidney replacement therapy is not made by a single physician but emerges through a hierarchical consultation process involving internal medicine residents, nephrology fellows, and senior academic nephrologists. This hierarchical clinical training model remains a cornerstone of postgraduate medical education, allowing internal medicine residents to develop clinical reasoning skills through supervised decision making. However, increasing patient volumes, time constraints, and variability in training experiences have stimulated interest in artificial intelligence-based decision-support tools that may assist internal medicine residents during the consultation process.

Large language models such as ChatGPT have demonstrated promising performance across a variety of medical knowledge and clinical reasoning tasks, yet their potential role within real-world nephrology consultation workflows remains largely unexplored. In particular, it remains unclear whether ChatGPT can accurately identify hemodialysis indications from nephrology consultation notes and reproduce dialysis-related decisions made in routine clinical practice. Although recent studies have reported that ChatGPT demonstrates high sensitivity in identifying patients at risk of requiring dialysis in acute kidney injury (10), evidence regarding its ability to generate a binary hemodialysis decision (yes/no) and identify specific dialysis indications based on real-world nephrology consultation notes remains limited. Furthermore, studies directly comparing these outputs with KDIGO-based recommendations, blinded expert nephrologist assessments, and actual clinical decisions are scarce.

The aim of this exploratory study was to evaluate whether ChatGPT can reproduce dialysis-related decisions made in routine clinical practice and to assess how closely its recommendations align with expert nephrologist consensus, thereby providing insights into both the clinical utility of LLMs and their potential value as educational support tools in specialist training programs.

## 2 Materials and methods

### 2.1 Study design and setting

This retrospective exploratory observational study compared the hemodialysis decision-making performance of ChatGPT with that of nephrology fellows and senior academic nephrologists. The analysis was based on archived nephrology consultation notes obtained from routine inpatient care at a tertiary care university hospital. The study protocol was reviewed and approved by the Akdeniz University Medical Scientific Research Ethics Committee (Decision No: TBAEK-433; approval date: 30 April 2026) prior to data collection and analysis. Because of the retrospective and non-interventional design of the study, the requirement for written informed consent was waived by the ethics committee. All data were fully de-identified before analysis, and no patient-identifiable information was shared with ChatGPT or external reviewers. The study was conducted in accordance with the principles of the Declaration of Helsinki and relevant national regulations on patient confidentiality and data protection.

### 2.2 Case selection

Patients who underwent nephrology consultation between March and April 2026 because of kidney function impairment were eligible for inclusion. Cases were included if complete consultation notes and corresponding clinical records were available for review.

Patients were excluded if consultation records were incomplete, unavailable, or lacked sufficient clinical information to determine whether hemodialysis was indicated.

Archived nephrology consultation notes were retrieved from the electronic medical record system. All records were anonymized before analysis by removing patient identifiers. Original consultation notes were used without modification to preserve the authenticity of the clinical information available during the initial consultation process.

### 2.3 Clinical consultation workflow: senior academic nephrologist and real-world clinical evaluation

**Figure 1:**
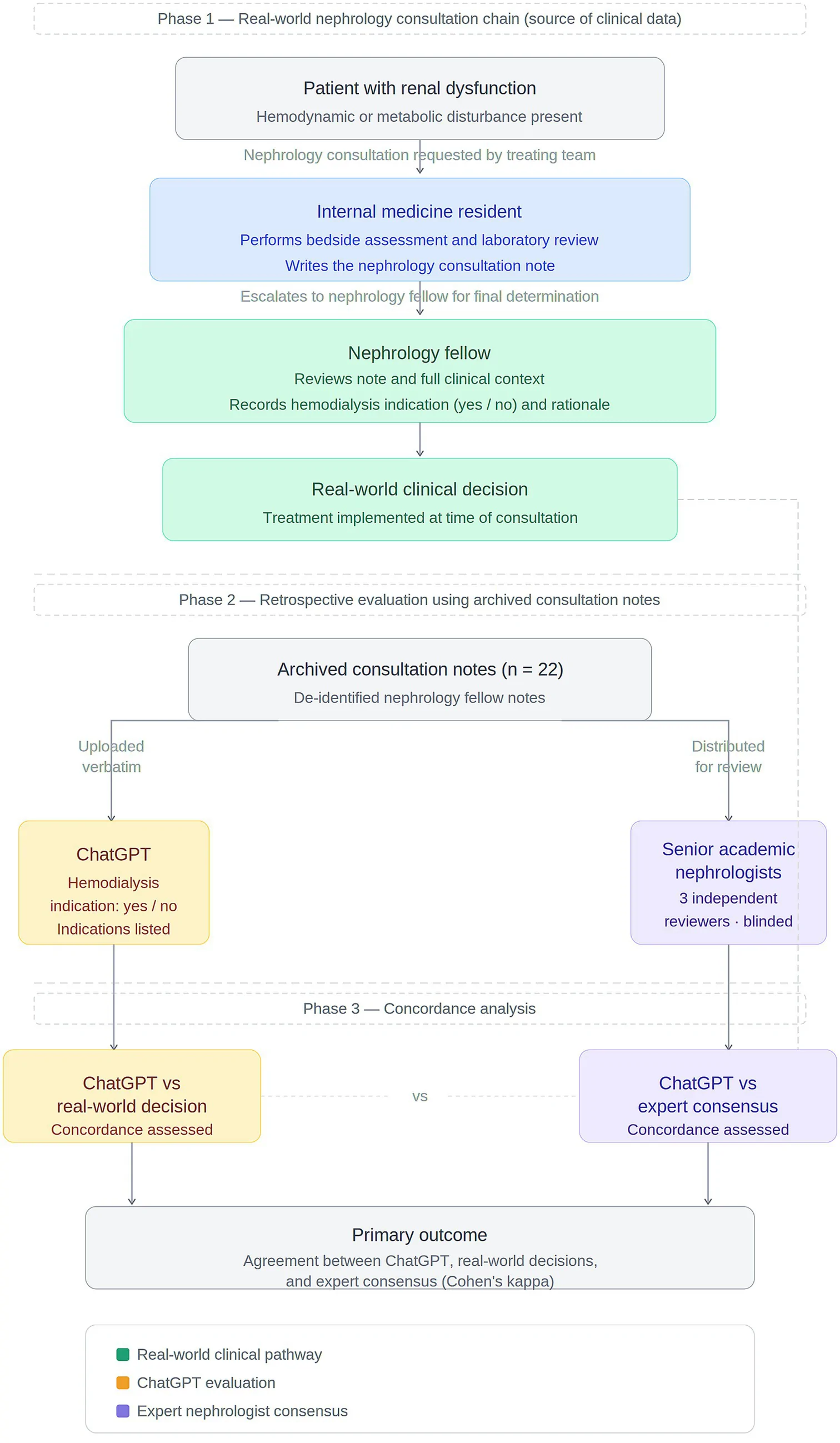
Study design and evaluation framework. The upper panel illustrates the routine nephrology consultation workflow at our institution, which served as the source of archived consultation notes and real-world clinical decisions. The lower panel depicts the retrospective study workflow, in which de-identified consultation notes were independently evaluated by ChatGPT and three senior academic nephrologists. Agreement between ChatGPT, real-world clinical decisions, and expert consensus decisions was subsequently assessed.

This retrospective study was based on archived nephrology consultation notes obtained from the routine consultation workflow of our institution. In daily clinical practice (Figure 1), nephrology consultations are requested for hospitalized patients with renal dysfunction, acute kidney injury, hemodynamic instability, metabolic disturbances, or suspected need for renal replacement therapy.

According to the institutional workflow, the initial evaluation is performed by an internal medicine resident. The resident reviews the patient’s clinical status, laboratory findings, and relevant medical records and documents a nephrology consultation note in the electronic medical record system. The case is subsequently discussed with a nephrology fellow, who reviews the consultation note together with the available clinical information and provides the final nephrology recommendation. This recommendation includes the determination of whether hemodialysis is indicated and guides patient management. Therefore, the nephrology fellow’s recommendation was considered the real-world clinical decision in the present study (Figure 1).

For study purposes, archived consultation notes were retrieved from the electronic medical record system and de-identified before analysis. The original consultation notes were used without modification. Each consultation note was independently evaluated by ChatGPT and by three senior academic nephrologists. Experts were blinded to the original clinical decision and to each other’s assessments.

The consultation notes were independently reviewed by three senior academic nephrologists with extensive clinical and academic experience in nephrology. Each nephrologist completed a standardized evaluation form consisting of the following items: (1) presence of a hemodialysis indication (Yes/No); (2) indication(s) for hemodialysis, including refractory hyperkalemia, metabolic acidosis, uremic symptoms (e.g., encephalopathy or pericarditis), fluid overload/pulmonary edema, oliguria/anuria, or other specified indications; and (3) optional additional comments. Reviewers based their assessments solely on the information available in the consultation note.

The nephrologists performed their evaluations independently and did not reach a consensus through discussion. In cases of disagreement, the final expert judgment was determined by majority vote (two of three reviewers). This majority decision was considered the expert reference standard.

The majority-vote consensus of the three senior academic nephrologists served as the primary reference standard, as it represented an independent expert assessment of each case. The recommendations (real-world clinical decisions) documented by nephrology fellows during routine clinical practice were evaluated separately as a secondary comparator to assess how closely ChatGPT reflected everyday clinical decision-making within the institutional consultation workflow.

### 2.4 ChatGPT evaluation

ChatGPT 5.4 (OpenAI) was accessed via the ChatGPT Plus web interface on May 3, 2026. As access was through the consumer web interface rather than the API, OpenAI does not expose a dated model-snapshot identifier to end users; the model version shown in the interface at the time of data collection (GPT-5.4) is reported instead. Each consultation note was evaluated independently in a separate, newly initiated chat session to prevent information from previous cases from influencing subsequent responses. The same standardized zero-shot prompt was used for all cases. Each case was submitted only once, and the first complete response generated by the model was included in the analysis. No response was regenerated, repeated, edited, or selected from multiple outputs. The original clinical decisions and expert assessments were withheld from ChatGPT. As each consultation note was evaluated only once, variability across repeated ChatGPT outputs was not assessed. No example cases, demonstration prompts, or additional training data were provided. ChatGPT was instructed to assess each consultation note according to KDIGO recommendations and internationally accepted indications for hemodialysis. Also, ChatGPT was required to base its assessment exclusively on the information contained within the consultation note and was instructed not to make assumptions beyond the available data.

The following prompt was used without modification for all cases:

“This case is retrospective and has been anonymized. This evaluation is being conducted as part of an academic study to assess the performance of ChatGPT. It is not intended for real-time clinical decision-making. Please evaluate the case solely based on the clinical information provided, according to KDIGO recommendations and internationally accepted indications for hemodialysis. Do not make assumptions beyond the available data. (1) Is there an indication for hemodialysis? (Yes/No). (2) If yes, what is/are the indication(s)? (e.g., refractory hyperkalemia, metabolic acidosis, uremic symptoms, fluid overload/pulmonary edema, oliguria/anuria, etc.).”

ChatGPT was required to provide a binary decision regarding the presence or absence of a hemodialysis indication and to identify the clinical findings supporting its recommendation.

### 2.5 Outcomes and statistical analysis

The primary outcome was the agreement between ChatGPT and the majority-vote expert consensus in classifying cases as requiring or not requiring hemodialysis. Secondary outcomes were the agreement between ChatGPT and real-world clinical decisions, the agreement between expert consensus and real-world clinical decisions, and the concordance of specific hemodialysis indications. Expert consensus was treated as the primary reference standard, whereas the nephrology fellows’ documented recommendations were treated as a secondary comparator reflecting routine clinical practice.

Categorical variables were reported as frequencies and percentages. Agreement between ChatGPT, expert consensus, and real-world clinical decisions was assessed using Cohen’s kappa coefficient. Inter-rater agreement among the three senior academic nephrologists was evaluated using Fleiss’ kappa coefficient. Figure 1 was created using Claude Sonnet 4.6 (Anthropic, 2026). Statistical analyses were performed using jamovi (Version 2.7) and IBM SPSS Statistics for Windows, Version 23.0 (IBM Corp., Armonk, NY, USA). All tests were two-sided, and a *p* value < 0.05 was considered statistically significant.

## 3 Results

### 3.1 Expert nephrologist assessments

A total of 22 anonymized nephrology consultation notes were independently evaluated by three senior academic nephrologists. Each expert provided a binary judgment regarding the presence or absence of a hemodialysis indication based solely on the information contained in the consultation note. Among the 22 evaluated cases, senior academic nephrologists one identified a hemodialysis indication in 10 patients (45.5%) and no indication in 12 patients (54.5%). Senior academic nephrologists two identified a hemodialysis indication in nine patients (40.9%) and no indication in 13 patients (59.1%). Similarly, senior academic nephrologists three identified a hemodialysis indication in nine patients (40.9%) and no indication in 13 patients (59.1%). Agreement among the three experts was high. Complete agreement among all reviewers was observed in 19 of 22 cases (86.4%), whereas disagreement occurred in only three cases (13.6%). No case required exclusion from the analysis due to unresolved disagreement. The majority-vote expert consensus classified nine patients (40.9%) as having a hemodialysis indication and 13 patients (59.1%) as not requiring hemodialysis. Inter-rater reliability analysis demonstrated excellent consistency among nephrologists. The Fleiss’ kappa coefficient was 0.814 (*p* < 0.001; Table 1).

**Table 1:** Distribution of hemodialysis decisions by senior academic nephrologists.

Assessment (cases = 22)	HD, *n* (%)	Non-HD, *n* (%)
Senior academic nephrologist 1	10 (45.5)	12 (54.5)
Senior academic nephrologist 2	9 (40.9)	13 (59.1)
Senior academic nephrologist 3	9 (40.9)	13 (59.1)
Expert consensus	9 (40.9)	13 (59.1)
Complete agreement	19 (86.4%)	
Disagreement	3 (13.6%)	
Fleiss’ kappa (95% CI), *p*-value	0.814 (0.562–1.000), <0.001	

CI, confidence interval.

### 3.2 Agreement between ChatGPT, expert consensus, and real-world clinical decisions

ChatGPT and real-world clinical decisions agreed on 18 of 22 cases, including 10 cases classified as requiring hemodialysis and eight cases classified as not requiring hemodialysis, resulting in an overall accuracy of 81.8% (*κ* = 0.633, *p* = 0.003). Four cases were classified differently. Agreement between ChatGPT and expert consensus was observed in 15 of 22 cases, including seven HD and eight non-HD classifications (accuracy 68.2%; *κ* = 0.374, *p* = 0.069). Disagreement occurred in seven cases. Expert consensus and real-world clinical decisions agreed in 17 of 22 cases, including eight HD and nine non-HD classifications (accuracy 77.3%; *κ* = 0.553, *p* = 0.007). Five cases showed discordant classifications (Table 2).

**Table 2:** Agreement between ChatGPT, expert consensus, and real-world clinical decisions.

Comparison	HD/HD, *n*	Non-HD/Non-HD, *n*	Disagreement, *n*	Accuracy (%)	Kappa (*κ*; 95% CI)	*p*
ChatGPT vs. expert consensus (primary comparison)	7	8	7	68.2	0.374 (0.004–0.744)	0.069
ChatGPT vs. real-world clinical decision (secondary comparison)	10	8	4	81.8	0.633 (0.308–0.958)	0.003
Expert consensus vs. real-world clinical decision	8	9	5	77.3	0.553 (0.220–0.886)	0.007

CI, confidence interval; HD/HD, both classified as requiring HD; Non-HD/Non-HD, both classified as not requiring HD; Disagreement, discordant classifications.

### 3.3 Case-level concordance of hemodialysis indications

Among the 22 cases, expert consensus classified nine cases as HD and 13 cases as non-HD. ChatGPT agreed with the expert HD decision in seven of nine cases. In all seven of these concordant HD cases, ChatGPT identified at least one expert-defined HD indication. Exact indication-level agreement was observed in four of seven cases. In the remaining three cases, ChatGPT either missed one expert-defined indication or suggested additional indications beyond the expert consensus.

According to real-world clinical decisions, hemodialysis was recommended in 12 cases. ChatGPT also recommended hemodialysis in 12 cases, and both approaches reached the same HD decision in 10 cases. In all 10 cases, ChatGPT identified at least one indication that overlapped with the real-world indication. Exact indication-level agreement was observed in four of 10 cases. The remaining cases showed partial overlap, mostly because ChatGPT assigned additional indications.

ChatGPT classified five expert-defined non-HD cases (Case 10, Case 11, Case 12, Case 14, and Case 22) as HD. These additional HD recommendations were most commonly associated with metabolic acidosis, oliguria/anuria, and fluid overload/pulmonary edema. Among the five expert-defined non-HD cases that ChatGPT classified as HD, three cases (Case 10, Case 11, and Case 12) involved lactic acidosis accompanied by reduced blood pH and were consistently classified by ChatGPT as metabolic acidosis-related indications for hemodialysis.

Compared with real-world clinical decisions, ChatGPT classified two real-world non-HD cases as HD, both involving fluid overload/pulmonary edema.

Conversely, ChatGPT classified two expert-defined HD cases as non-HD. The missed expert indications were fluid overload/pulmonary edema in both cases and oliguria/anuria in one case. Compared with real-world clinical decisions, ChatGPT classified two real-world HD cases as non-HD, involving oliguria/anuria and metabolic acidosis (Table 3).

**Table 3:** Case-by-case comparison of hemodialysis decisions and indications among expert consensus, real-world clinical decisions, and ChatGPT.

Case	Expert decision	Expert indication	Real-world decision	Real-world indication	ChatGPT decision	ChatGPT indication	Explanation of discordance
Case 1	HD	5, 4, 2, 1	HD	5, 4, 2, 1	HD	5, 2, 1	ChatGPT missed fluid overload/pulmonary edema
Case 2	HD	5, 4	HD	5	HD	5, 4	None
Case 3	HD	5, 4	Non-HD	—	Non-HD	—	Expert HD indication missed by both real-world decision and ChatGPT
Case 4	HD	5	HD	5, 4	HD	5	None
Case 5	HD	4	HD	5	Non-HD	—	ChatGPT missed expert HD indication
Case 6	HD	4	HD	4	HD	4	None
Case 7	HD	4	HD	4	HD	4	None
Case 8	HD	2	HD	5, 4, 2	HD	5, 4, 2	ChatGPT suggested additional indications beyond expert consensus
Case 9	HD	2	HD	2	HD	5, 3, 2	ChatGPT suggested additional indications beyond expert consensus
Case 10	Non-HD	—	HD	5, 2	HD	5, 2	HD recommended despite expert non-HD consensus
Case 11	Non-HD	—	HD	5	HD	5, 4, 2	HD recommended despite expert non-HD consensus
Case 12	Non-HD	—	HD	5	HD	5, 2	HD recommended despite expert non-HD consensus
Case 13	Non-HD	—	HD	2	Non-HD	—	Real-world HD despite expert non-HD consensus
Case 14	Non-HD	—	Non-HD	—	HD	5, 4	ChatGPT false-positive HD recommendation
Case 15	Non-HD	—	Non-HD	—	Non-HD	—	None
Case 16	Non-HD	—	Non-HD	—	Non-HD	—	None
Case 17	Non-HD	—	Non-HD	—	Non-HD	—	None
Case 18	Non-HD	—	Non-HD	—	Non-HD	—	None
Case 19	Non-HD	—	Non-HD	—	Non-HD	—	None
Case 20	Non-HD	—	Non-HD	—	Non-HD	—	None
Case 21	Non-HD	—	Non-HD	—	Non-HD	—	None
Case 22	Non-HD	—	Non-HD	—	HD	4	ChatGPT false-positive HD recommendation

1: Refractory hyperkalemia; 2: Metabolic acidosis; 3: Uremic symptoms; 4: Fluid overload/pulmonary edema; 5: Oliguria/anuria.

Shaded rows indicate cases in which the overall hemodialysis decision (HD vs. Non-HD) was discordant between ChatGPT and either the expert consensus or the real-world clinical decision.

## 4 Discussion

This study evaluated the ability of ChatGPT to identify hemodialysis indications from real-world nephrology consultation notes and examined the agreement between its recommendations, real-world decisions made by nephrology fellows, and the consensus of senior academic nephrologists. ChatGPT demonstrated moderate agreement with fellow-level decisions (accuracy 81.8%; *κ* = 0.633, *p* = 0.003), whereas agreement with expert consensus was lower and did not reach statistical significance (accuracy 68.2%; *κ* = 0.374, *p* = 0.069). These exploratory findings suggest that ChatGPT showed greater agreement with the real-world decisions of nephrology fellows than with the consensus of senior academic nephrologists. This observation may reflect differences in clinical experience and decision-making approaches between the two groups. Unlike previous studies (10, 11), which evaluated ChatGPT using risk scores or included only patients who had already undergone dialysis, our study used authentic nephrology consultation notes from both HD and non-HD patients. This design provided a more realistic evaluation of its ability to support hemodialysis decision-making in routine clinical practice.

The performance of LLMs across medical decision-making tasks has been the subject of growing investigation. Prior studies have demonstrated that ChatGPT can achieve passing scores on standardized licensing examinations, generate clinically plausible differential diagnoses, and provide guideline-concordant management summaries across a range of specialties (7, 12, 13). In addition, ChatGPT has also been evaluated in patient counseling and medication-related information tasks, where it was found to provide scientifically appropriate and understandable information to patients (14). In nephrology, LLMs have shown promising performance in interpreting laboratory findings, triaging consultation requests, and managing continuous renal replacement therapy alarms (4, 5). However, most studies have relied on knowledge-based benchmarks or simulated clinical scenarios rather than authentic consultation notes (15). Two recent studies evaluated ChatGPT in dialysis decision-making. Can et al. reported a high sensitivity of 94–97% for identifying patients with AKI who required dialysis (10). However, nephrologists in that study rated ChatGPT’s responses using a Likert scale and did not provide independent yes/no decisions. Kara et al. reported 100% agreement with nephrologist decisions, but their cohort included only patients who had already been selected for dialysis, preventing evaluation of false-positive recommendations (11). In contrast, our study included both patients with and without indications for dialysis. This allowed evaluation of not only agreement and sensitivity but also specificity and false-positive decisions. The present study addresses this gap by evaluating ChatGPT on de-identified consultation notes produced prospectively within a real institutional workflow, thereby introducing the complexity and variability inherent to actual clinical documentation. To our knowledge, this is among the first studies to compare ChatGPT outputs with both a primary reference standard based on blinded senior expert consensus and a secondary comparator based on real-world decisions made by nephrology fellows within the same dataset. Although majority-vote expert consensus was used as the primary reference standard, expert judgment is not an absolute ground truth and may vary according to clinical experience and interpretation. The real-world clinical decisions were therefore retained as a secondary comparator to reflect routine practice. The institutional consultation workflow, in which internal medicine residents prepare consultation notes that are subsequently reviewed by nephrology fellows, provided a realistic setting for evaluating ChatGPT within the educational structure of nephrology training.

The moderate agreement between ChatGPT and real-world clinical decisions (*κ* = 0.633) is consistent with previous studies evaluating LLM performance in nephrology. Can et al. reported a sensitivity of 94%–97% for identifying patients with acute kidney injury who required dialysis and found a high degree of concordance between ChatGPT outputs and nephrologist assessments (10). Similarly, Chen et al. reported moderate to substantial agreement between LLM recommendations and physician decisions across several nephrology scenarios (6). A possible explanation for the agreement observed in our study relates to the consultation process itself. Both ChatGPT and the nephrology fellow based their decisions on the same consultation note, and this shared information source may have contributed to the observed level of agreement. These findings suggest that ChatGPT may be particularly useful in consultation-based settings where decisions rely heavily on documented clinical summaries. Notably, the agreement observed in the present study was achieved using a more rigorous comparison framework than that used by Can et al. (10). In that study, ChatGPT outputs were rated by nephrologists, whereas our study compared ChatGPT directly with independent clinical decisions made during routine practice.

ChatGPT showed lower agreement with senior academic nephrologists than with nephrology fellows, which may reflect the difference in clinical experience between these two groups. Fellows, still developing their clinical judgment, tend to rely heavily on documented findings and guideline-based reasoning. This approach resembles how ChatGPT generates recommendations from information explicitly stated in the consultation note and may explain the higher concordance observed between them. Senior nephrologists, by contrast, draw on years of accumulated experience, pattern recognition, and clinical intuition when managing complex or atypical presentations (16, 17). This is consistent with prior work showing that LLMs perform well on structured, guideline-driven tasks but less reliably when decisions require contextual interpretation and expert judgment (8, 9, 12, 18). Taken together, these findings suggest that the disagreement between ChatGPT and expert consensus reflects not only differences in recognizing clinical findings but also in how those findings are interpreted within the broader clinical course.

An additional finding that may help explain the discordance between ChatGPT and the consensus of senior academic nephrologists relates to the interpretation of metabolic acidosis, volume overload, and oliguria/anuria. Three cases classified as non-HD by the expert panel were identified by ChatGPT as requiring hemodialysis because of metabolic acidosis. All three cases involved lactic acidosis accompanied by a reduced blood pH. While ChatGPT appeared to interpret acidemia as a direct indication for dialysis, experienced nephrologists may have considered the underlying mechanism and potential reversibility of the acid–base disturbance (19). In clinical practice, lactic acidosis is often initially managed by addressing tissue hypoperfusion and monitoring the response to medical treatment before escalation to renal replacement therapy (19–21). A similar pattern was observed in two cases with clinical findings suggestive of volume overload, including peripheral edema and pulmonary crackles. ChatGPT interpreted these findings as indications for hemodialysis, whereas the expert consensus favored continued medical management and reassessment of the response to diuretic therapy. This limitation aligns with recent findings by Kara et al., who reported that while ChatGPT demonstrated perfect agreement with nephrologists on the initial decision to dialyze, its concordance significantly decreased regarding ultrafiltration parameters. They explained this difference by noting that managing a patient’s fluid levels requires real-time observation, which an AI cannot do just by reading fixed numbers on a screen (11). Likewise, in four cases classified as non-HD by the senior academic nephrologists, ChatGPT identified oliguria/anuria as a sufficient indication for hemodialysis. This finding suggests that ChatGPT may place greater emphasis on reduced urine output itself, whereas experienced nephrologists are more likely to interpret oliguria within the broader clinical context. In routine practice, the decision to initiate hemodialysis is not based solely on urine output but also on volume status, metabolic complications, response to medical management, and the overall clinical trajectory (22). Taken together, these observations suggest that ChatGPT’s reasoning is anchored to the static clinical findings documented in the note, whereas experienced nephrologists additionally draw on treatment response, disease trajectory, and bedside clinical experience. This reflects a fundamental limitation of current generative AI models in time-sensitive clinical contexts (23, 24).

The threshold for initiating renal replacement therapy is known to vary across institutions, training backgrounds, and individual physicians (3, 25). The moderate agreement observed between expert consensus and real-world clinical decisions in the present study further highlights this variability (26). These findings raise a fundamental methodological challenge in studies evaluating clinical decision-making: when expert consensus and routine clinical practice diverge, neither can be uncritically accepted as a single ground truth (27, 28). Previous studies have typically relied on a single reference standard. Kara et al. used nephrologist prescribing decisions, whereas Can et al. evaluated ChatGPT outputs through expert review (10, 11). By incorporating both real-world fellow-level decisions and an independent consensus of three senior academic nephrologists, the present study provides a broader assessment of clinical agreement and reflects the inherent variability of medical decision-making. Future studies should explicitly address this issue and consider reporting agreement against multiple reference standards simultaneously.

The findings of this study carry important implications for the role of AI in nephrology training. ChatGPT demonstrated greater agreement with fellow-level decisions than with the consensus of senior academic nephrologists, suggesting that its reasoning may more closely resemble the approach of nephrology fellows who are still developing advanced clinical judgment. The observed discrepancies indicate that ChatGPT can identify clinically relevant findings but may not consistently distinguish between abnormalities that warrant immediate renal replacement therapy and those that require continued monitoring, medical management, or reassessment over time. Consequently, ChatGPT may have potential as a supervised educational tool by encouraging clinicians in training to systematically review dialysis indications and reflect on their clinical decisions,an approach broadly supported by recent randomized controlled trials showing that LLM-generated feedback and simulated clinical encounters can improve clinical reasoning among medical trainees (29, 30). Because the educational impact of this approach was not evaluated in the present study, whether it translates into improved clinical reasoning or educational performance remains unknown and warrants evaluation in prospective studies.

### 4.1 Limitations

Several limitations of the present study must be acknowledged. The sample of twenty-two consultation notes, drawn from a single tertiary care institution, limits the statistical precision of the agreement estimates and precludes generalization to settings with different consultation documentation practices or patient populations. The single-center design also means that the real-world decisions reflect the clinical culture and threshold practices of one nephrology training program, which may not be representative of broader practice. Accordingly, the present findings should be regarded as exploratory and hypothesis-generating, providing a foundation for larger prospective validation studies. ChatGPT was evaluated using a single model version with a standardized prompt; performance may differ across model iterations, prompt formulations, or when additional clinical context is provided. Furthermore, each consultation note was evaluated only once. Therefore, the study did not assess within-model response variability or the consistency of ChatGPT outputs across repeated evaluations of the same case. Future research should address these limitations through multicenter studies with larger and more diverse consultation note datasets, repeated independent evaluations to assess the stability and reproducibility of model-generated decisions, head-to-head comparisons of multiple LLM platforms under identical conditions, and prospective educational studies evaluating whether LLM-assisted learning improves clinical reasoning during nephrology training.

## 5 Conclusion

In this preliminary study, ChatGPT demonstrated moderate agreement with nephrology fellow decisions but lower agreement with the consensus of senior academic nephrologists when evaluating real-world nephrology consultation notes for hemodialysis indications. Discrepancies were most evident in cases involving metabolic acidosis, volume overload, and oliguria/anuria, where expert decisions incorporated clinical context, treatment response, and disease trajectory beyond the information documented in the consultation note. A key strength of this study was the use of real-world nephrology consultation notes from both HD and non-HD cases, together with a primary expert reference standard, comparison with real-world clinical decisions, and indication-level analysis, allowing a more detailed evaluation of ChatGPT’s clinical reasoning than has been reported in previous nephrology studies. These findings suggest that ChatGPT should not be considered a substitute for expert clinical judgment. However, these preliminary findings suggest that ChatGPT may have potential to support decision-making during nephrology training. Although these consultation notes reflected real-world practice, future consultation documentation may be further standardized with potential LLM integration in mind. More structured clinical summaries could improve the quality and consistency of AI-generated outputs. Future studies should evaluate whether AI systems can be integrated into real-time consultation processes and support supervised decision-making within the traditional educational hierarchy of medicine.

## Data Availability

The data analyzed in this study is subject to the following licenses/restrictions: De-identified data may be available from the corresponding author upon reasonable request and subject to institutional and ethical approval requirements. Requests to access these datasets should be directed to drdilekyapar@outlook.com.
